# SISME, Estuarine Monitoring System Based on IOT and Machine Learning for the Detection of Salt Wedge in Aquifers: Case Study of the Magdalena River Estuary

**DOI:** 10.3390/s21072374

**Published:** 2021-03-29

**Authors:** Paola Patricia Ariza-Colpas, Cristian Eduardo Ayala-Mantilla, Qaisar Shaheen, Marlon Alberto Piñeres-Melo, Diego Andrés Villate-Daza, Roberto Cesar Morales-Ortega, Emiro De-la-Hoz-Franco, Hernando Sanchez-Moreno, Butt Shariq Aziz, Mehtab Afzal

**Affiliations:** 1 Department of Computer Science and Electronics, Universidad de la Costa CUC, Barranquilla 080002, Colombia; rmorales1@cuc.edu.co (R.C.M.-O.); edelahoz@cuc.edu.co (E.D.-l.-H.-F.); 2 Faculty of Marine Sciences, Escuela Naval de Suboficiales ARC “Barranquilla”, Armada Nacional de Colombia, Barranquilla 080002, Colombia; cristian.ayala@armada.mil.co (C.E.A.-M.); diego.villate@armada.mil.co (D.A.V.-D.); 3 Department of Computer Science, Superior College, Lahore 44000, Pakistan; qaisar.shaheen2002@gmail.com; 4 Department of Systems Engineering, Universidad del Norte, Barranquilla 081001, Colombia; pineresm@uninorte.edu.co; 5 Facultad de Ciencias Básicas, Universidad Simón Bolívar, Centro de Investigación e Innovación en Ciencias Marinas y Limnológicas del Caribe Colombiano “CICMAR”, Barranquilla 080001, Colombia; 6 Department of Computer Science and IT, University of Lahore, Lahore 44000, Pakistan; shariq2315@gmail.com (B.S.A.); mehtab.afzal@cs.uol.edu.pk (M.A.)

**Keywords:** IOT systems, machine learning, salt wedge, aquifers, Magdalena river estuary

## Abstract

This article contains methods, results, and analysis agreed for the development of an application based on the internet of things and making use of machine learning techniques that serves as a support for the identification of the saline wedge in the Magdalena River estuary, Colombia. As a result of this investigation, the process of identifying the most suitable telecommunications architecture to be installed in the estuary is shown, as well as the characteristics of the software developed called SISME (Estuary Monitoring System), and the results obtained after the implementation of prediction techniques based on time series. This implementation supports the maritime security of the port of Barranquilla since it can support decision-making related to the estuary. This research is the result of the project “Implementation of a Wireless System of Temperature, Conductivity and Pressure Sensors to support the identification of the saline wedge and its impact on the maritime safety of the Magdalena River estuary”.

## 1. Introduction

The design of monitoring networks is a topic that has received considerable attention within hydrological literature. Monitoring networks with IOT in general aim to quantify a variable that varies in space and time using a limited number of stations measurement that must be distributed in the observation region in an optimal way, to capture the spatial variability of the variable of interest in the best possible way [1]; it is therefore essential that there be judicious studies that allow optimizing resources and of course that guarantee the correct achievement of the data collection as the main objective of an installation.

In an ideal design of a monitoring network with IOT, a comprehensive answer should be given to questions how, where, when, and how much to monitor, as well as the evaluation of network effectiveness to meet future information needs [2], so it is necessary to be careful with the planning. That is why it is very important to have a planning process that guarantees the correct operation of the system to be installed.

One of the main problems faced when installing measuring equipment is the need to identify what will be covered with it, since every monitoring network must be designed to meet goals or objectives [3], which depend on the needs raised in the project that allows for its execution. In the network target for monitoring applied for this study are two important factors that are surveillance related to the study of variability of the temperature, salinity, pressure, and level present in a section of the Magdalena River, an objective that is also nested in the possible development of a model prediction of the behavior of these variables, which makes it necessary to identify the areas where it is assumed that the variables to be measured will have a higher incidence or capture factor.

Another aspect to consider is the change of objectives that a monitoring network can experience. A network may have been established for a specific project and over the years these goals may have changed. As we mentioned earlier, the cost and maintenance aspect of networks cannot be ignored and this leads to the design of monitoring networks by developing an optimal objective function [4]. Taking advantage of what is installed is a very important aspect because the location allows us to know the geographical and environmental aspects of the study area.

The most important contributions of this work are having for the first time a system that can transmit real-time information concerning the state of the study variables, such as: temperature, pressure, and conductivity in the estuary of the Magdalena river. The results obtained when processing this information using machine learning techniques such as Multi-Layered Perceptron are shown, applying time series for processing. The reliability of the model and the congruence of the results can be evidenced in later sections by contrasting the mean square error and the accuracy.

This article is organized in the following way. First, a brief review of the literature referenced is described (Section 2). Second, the climate conditions of the area of the study are described (Section 3). Third, phases for the development of the SISME software are specified (Section 4). Fourth, a contribution is shown (Section 5). Finally, the results of the implementation, the conclusions, and future works are shown (Section 6).

## 2. Literature Review

Monitoring systems based in IOT have become one of the highly important allies in the process of measurement, monitoring, and control of different variables of the ecosystem order. Many authors of recent research papers have exposed through their publications the various ways in which they can use this monitoring system to support decision-making regarding aspects of the biological and environmental order.

Miller [5] implemented a sensor network in Kachemak Bay, Alaska. Through the configuration and application of this monitoring network, it was possible to identify the dynamics of the pH and the existing variation between the carbon chemistry in the estuary, making use of time series-based analyses. Schrand [6] carried out research in the ecosystem in Tampa Bay, Florida, to be able to identify the different conditions of the animals existing in the aquifer; for them, the interaction between both fresh and salty waters is considered.

Lin [7] conducts research in marine protected areas in the Guangxi Coastal Wetlands Nature Reserve, China. This application shows its implementation advantage because many solutions that existed on the market did not respond to the characteristics of the body of water. This application shows four important benefits, namely: (1) It could have indicators that allowed to analyze the behavior of the wetland from the ecological, social, and economic point of view. Unlike supervision, (2) it is based on indicators that define the condition of the wetland itself, which provides valuable support for decision-making. (3) It has a weighting system that allows the results to be totally objective compared to the current state of the estuary. This is to minimize the influence of subjective factors. (4) The monitoring system provided statistical data to support decision-making by the local government. Other authors such as Krunal Patel [8] make use of satellite images to be able to carry out multitemporal analyses and identify the changes that are caused by different factors such as anthropogenic activities, the rise in sea level, and erosion.

Vieira [9] carries out studies in the Vitória Bay in Brazil. The purpose of his research is focused on the identification of metals such as Cd, Pb, Cu, Ni, and Fe, which are found in this body of water and which in turn, when found in concentrations that are not allowed, can have health consequences for permeating the generation of Cancer in the population. Han [10] conducts research in the Yellow River delta in China. This place was selected for the study because as it is considered a coastal wetland, it consists of a wide diversity of species that, when housed in it, constitute a valuable aspect for the ecosystem.

Chen [11] exposes in his article the consequences of the affectation of the diverse human activities in the marine pollution. To analyze these factors, the results of the implementation of an ecological monitoring system have been presented, as part of an early warning system to carry out regulatory processes associated with the marine economy that do not directly affect the species. Hsieh [12] focused his research in the Kaomei estuary, Taiwan, implementing a monitoring system using infrared. The research focused on the analysis of the variation of greenhouse gas emissions in plants in and around the Kaomei estuary.

Other researchers have used the sensor network to analyze the conditions for the generation of different species in estuaries. Dale [13] implements sensors for the measurement of salinity, temperature, and oxygen (that is, water condition) in Apalachicola Bay, Florida (USA), to determine the conditions for the process of improving the habitat for the development of the Gulf sturgeon, which is in danger of extinction because currently, given the conditions of different bodies of water, it does not allow the conditions for its reproduction to be available. Barthelemy [14], in his article, shows the implementation of the Smart Stormwater Management project, which can identify the clogging of sewers in real time and manage the management of estuaries, to achieve water quality, identifying gross pollutants that can cause diseases in the population.

## 3. Relevant Characteristics of the Magdalena River Estuary

The Magdalena River runs approximately 1550 km from its source in the lagoon of La Magdalena, located in the Colombian massif, to the Caribbean Sea in Bocas de Ceniza [3] (Barranquilla) and the Canal del Dique (Cartagena). It has an average flow of 7100 m^3^/s at the height of Squid, before its fork in the Dique channel. The Magdalena is a typical example of rainwater river. The level of its waters shows appreciable variations due to the strong local rainfall, both on its riverbed and on its tributaries. These variations are more appreciable in its upper and middle sectors, since in lower Magdalena the regulation is exerted by the swampy zone, where, in addition to reducing rainfall, it accumulates large amounts of water during the rainy season, which are returned to the channel in the periods of low water or drought.

The variety, one of the main characteristics of the basin, acquires a special relevance in the field of climatology, since the combination of factors generates a huge number of environments subjected in many cases to strong alterations of the general patterns by the incidence of local characteristics, such as the shape and extent of the relief and the way in which this affects the rains and atmospheric circulation. However, IDEAM has proposed a division of the basin into climatic regions that, having made the previous exceptions, demonstrate certain homogeneity in its characteristics [4].

### 3.1. Climatology of the Magdalena River

The Magdalena Basin is located approximately between 2° and 11° N, in full tropical zone, which makes it present special characteristics [3]; for the case of the Lower Magdalena region, which is the area of our interest, the characteristic climate is the warm dry and wet type. To a lesser extent, very dry climates appear in extremes in the northwest and very humid climates in the south. Like the rest of the Caribbean, there are three climatic seasons:

- Dry season (December-March): There is a predominance of dry time and generally a reduction in relative humidity.
- Transition time (April-July): This time is produced by the weakening of the trade winds from the northeast and the displacement of the ITCZ to the north [15], which are presented winds with variable directions, coming mainly from the NE quadrant; 80% of the time, the wind speed does not exceed 8 m/s.

### 3.2. Approach to the Climate Conditions of the Area of Study

**Figure 1:**
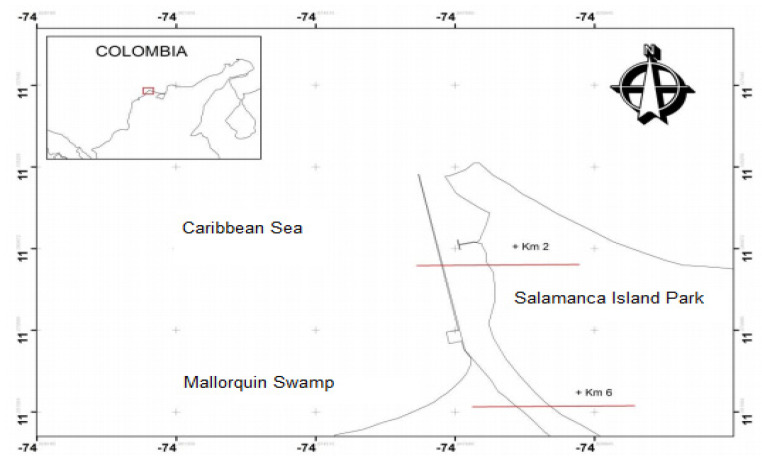
Study area location.

The study area is in the Department of Atlántico, on the final sector that gives the mouth of the Magdalena River on the Caribbean coast between the coordinates, see (Table 1 and Figure 1):

Next, the characteristics of the Magdalena River are shown, in terms of its climatic conditions:

- Temperature: The average temperature value registered in this area is 28° with maximums of up to 32 °C [16].
- Clouds: cover is closely linked to general climatic behavior, presenting variability of percentages with respect to the climatic season in which it is found. Dry season ≥ 0% (clear) most of the time. Transition Time ≥ 40% (cloudy) most of the time. Season Humidity ≥ 60% (Mostly cloudy).
- Precipitation: Rainfall begins in April with the beginning of the transition period, however, due to the poorly defined conditioning of the synoptic behavior, there are depressive and anticyclonic zones.
- Humidity: The proximity to the sea, the area of the Isla Salamanca National Natural Park, and the wetlands of the delta of the mouth of the Magdalena River, makes this area have high enough humidity levels, but this humidity is modified by the drying winds and pushes it towards the interior of the region to produce abundant rains in the foothills of the Andes.

### 3.3. General Fluviography of the Magdalena River

The planes of the continental shelf between Cartagena and Santa Marta are covered by thick terrigenous deposits of the Pliocene and Quaternary, which resulted from successive ramblings of the Magdalena River [17]. The Lower Magdalena Basin is not only extensive but deep, having an estimated thickness of Tertiary strata of more than 4000 m [18]. The plain or deltaic plane of the Magdalena River has been subdivided into five geomorphological units: [19,20]: River floodplain Magdalena; Lagoon system of the current delta; Marginal lagoon system; Beach and Barrier Island; and Piedmont alluvial plain.

### 3.4. General Characterization of the River

The Magdalena River and its floodplain. They are young and flat areas subject to overflows direct and periodic from the river [21], which include all forms of land originated by direct fluvial sedimentation processes (overflow, flood, decantation, deltaic arms, meadows, and banks). On the banks of the eastern side of the river, there is a general tendency to erosion (bank scour) and on the western side to deposition (low colonized by aquatic plants), already mentioned by Lorin [22]. According to these authors, the geomorphology of the banks and the bottom of the river is highly influenced by a paleorelieve where the rises of the basement and the proximity of quaternary levels play considerable a role. The floodplain presents geomorphological features such as banks, channels (a sometimes truncated), dikes, swamps, lagoons, and paleo-channels of the Magdalena. In accordance with [23], it is made up of silty muds and fine dark gray sands enriched with organic matter.

### 3.5. General River Hydrology

The Magdalena River contributes water to the deltaic plain due to superficial overflow at levels of relatively high waters. The cyclical increases of the Magdalena generate multi-year changes in the water levels of the lagoons [24]. Kaufmann and Hevert [25], found every 6–7 years maximum annual averages and every 6–7 years minimum annual averages of the river. In low and medium waters only, stagnation occurs and then evaporation progressively reduces the extension and depth of the swamps and temporary pipes. According to the flood levels, the direction of the current can be reversed and, especially the lagoons of the sector south and west can feed old Magdalena arms instead of receiving water from them.

### 3.6. Sediment Transport Processes

The sediments that are deposited in the lagoon system of the Magdalena deltaic plane are transported there by two means: water and air. Wind transport is concentrated in extensive dunes of the coastal area and can carry materials from this site to the interior lagoons. The water transport includes floods and flows of water through pipes from the river Magdalena; coastal transport with the intervention of waves, currents, and tides; direct transport from the Sierra Nevada de Santa Marta to the Cienega Grande by the western drainage of the mountainous system and reworking. The main sources of sediment for streams are coastlines of the area are the Magdalena River to the west, the Sierra Nevada to the east, deposits of marine quaternaries such as ancient deltaic lobes of the Magdalena River, and sediments of coastal areas from the east of the system.

### 3.7. Physical Characteristics of the Magdalena River and Its Relationship with the Caribbean Sea

According to what was studied by [26], the Magdalena delta can be classified as a mixed domain, influenced by fluvial inputs and waves (i.e., fluviowave dominated type). The same author identifies that with data measured at the Calamar hydrological station, this delta receives a flow of 205.1 km^3^ a −1 of water and 142.0 × 106 t a −1 of suspended sediments [26]. On average, the delta is influenced by the presence of bottom waves (i.e., swell) coming from the northeast, with significant heights (Hs) of 2.2 ± 1.1 m and a peak period of 6.7 ± 2.3 s [27]. The dry and transitional season, the saline wedge can have different effects reaching up to 4.54 km upstream from the mouth in the month of November, while in April the intrusion reached up to 6.94 km [26].

### 3.8. Considerations Related to the Present Climate Condition

Once the secondary information was studied, it was identified that the fluvial area where the hydrological and oceanographic monitoring network is to be installed has typical conditions that do not imply any major setback to the development of the project.

In relation to atmospheric conditions, the variables have permissible ranges that will not affect the equipment or the infrastructure to be installed, with respect to the river’s own fluvial conditions; it is necessary to indicate that they are not contrary to the provisions or ranges that the equipment that can be used can handle and intend to install. However, a conditioning that must be considered is the dynamics that the Saline Wedge may possibly have, mainly because the characteristics of an estuary and its degree of stratification are determined by two fundamental factors: the discharge of fresh water, which tends to maintain the stratification against saline water penetrating the lower course of the river, and the tide, which tends to produce turbulent mixing and consequently to reduce stratification. The preponderance of one of these factors over the other will define the type of estuary and the level of mixing and stratification that exists in the same.

## 4. Phases for the Development of the SISME Software

For the development of this solution, a set of phases was considered, which contemplated highly relevant aspects in the process of obtaining the software, which will be detailed below:

### 4.1. Phase 1: Sensor Location Identification

Considering that it is necessary to perform water column measurements to achieve the characterization of the desired variables, the location of the monitoring stations is the most critical factor to evaluate, even more so when experience indicates that the equipment left in areas not previously studied can be lost, be damaged or simply not take the data you want. Sanders, Adrián and Berger [28] recommend the location of stations according to some subdivisions among which are macro-location and micro-location; the first is a systematic process that leads to evaluate in a whole what could be considered capable of measuring while micro-location focuses on the idea that it is necessary to locate the equipment in critical places or identified as sensitive to the variables to be measured; in this sense, this sector has already been identified thanks to studies and related secondary information in the previous section.

In the case of the variables that need to be measured by the network that needs to be installed, it must be considered as an important point to know where the mixing ratio is regularly based on the flow outlets and inlets, which empirically and theoretically indicates that the ideal place is the first 6 km of the Magdalena river delta. In this sense, two options were evaluated that locate the sensors at the edge of the river bank (Port facility or station manufacturing) and the maritime signaling buoys.

The criteria were identified according to the needs, so for a better understanding an explanation of each one is made:

Accessibility for maintenance: This criterion focuses on the facilities to reach the equipment, the port facility being the one that fulfilled this condition in the greatest sense, since its access is by land.

Installation costs: For this criterion, the economic value that can include civil works or masonry works is considered, with buoys being the one that would imply the lowest cost since their structure would be used to locate them.

Data coverage in relation to spatial geographic conditions: This criterion should take into account what is described by the methodology proposed by Sanders, Adrián and Berger [28], so that placing the sensors in the center of the channel would give greater information records without disturbances or noises from external agents. In some consulted bibliography it was identified that the values of mixing or saline intrusion are better appreciated near the canal because of the effect of the flow; therefore, installing the equipment in riparian areas would not allow to characterize the water column in such an effective way.

Equipment security: Equipment security is something that certainly raises concern, especially due to vandalism that can generate ignorance of its usefulness; in a port facility this criterion is more than met due to 24-h security, while in port facilities, the two other options not so much. However, in the case of the buoy, its difficult accessibility, added to the fact that most of the equipment will be installed submerged, can generate some peace of mind regarding its protection.

#### Installing the Sensors on the Buoys

In Table 2, identify how far the 6 km goes and the buoys that can possibly be used as sensor installation platforms. Buoy 1 is the closest to the mouth so they would be considered first-hand as the one that can obtain data with higher resolution. To give a greater range of action to the counting of the teams, buoy 7, which is close to Kilometer 6, was also chosen.

**Figure 2:**
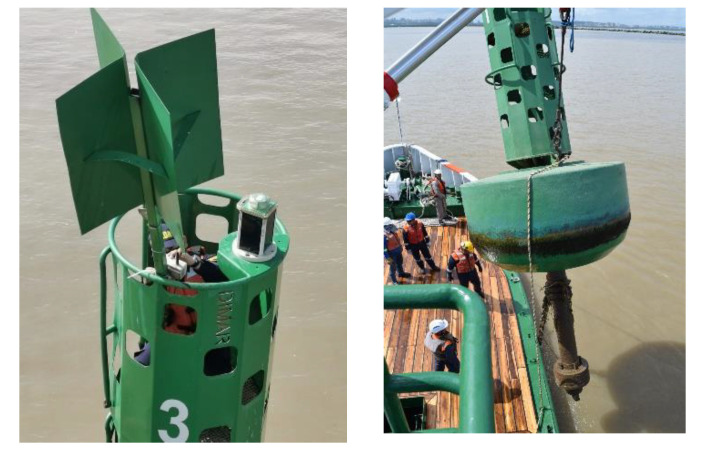
Installation process of the buoys with the sensor systems in the Magdalena River.

The buoys have a height of 8733 m from its base, the floating structure of that rises out of the water, gives an approximate total of 5621 m, and the width of the upper structure is 0.92 m. Figure 2 shows the installation process of the buoys with the sensor systems in the Magdalena River.

### 4.2. Phase 2: Tools Used for Software Development

**Figure 3:**
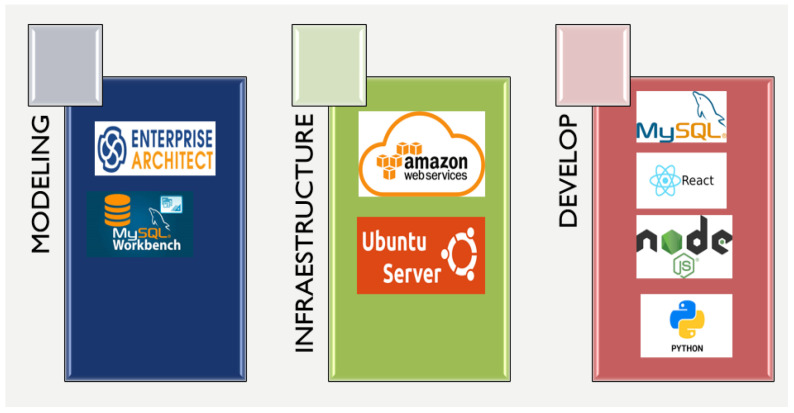
Tools used for the development of the platform.

To develop the application, a set of tools were used that made it possible to cover each of the phases of software development. Figure 3 shows the set of applications that served in the phases: modeling, infrastructure, and development.

In Figure 3 each of the tools used in the software construction phases is shown. In the modeling phase, Enterprise architect was used to be able to carry out the entire requirements of the engineering process based on the UML language; in the same way, MySQL Workbench was also used, which allows the design of the database to be defined graphically, also allowing the administration and maintenance of the database. In the infrastructure phase, considering the redundancy and data availability requirements, Amazon Web Services was implemented. As a cloud computing service, this tool was complemented with Ubuntu Server, as a basis for the data and information repository by making use of tools developed by the community of free software developers. Regarding the development phase, the use of Mysql was considered, as an engine and repository for the data coming from the buoys. The development of both the back-end and the front-end was done in the End React JS and End Node JS javascript language respectively. As for the programming language of the predictive model based on machine learning techniques, Python was selected.

**Figure 4:**
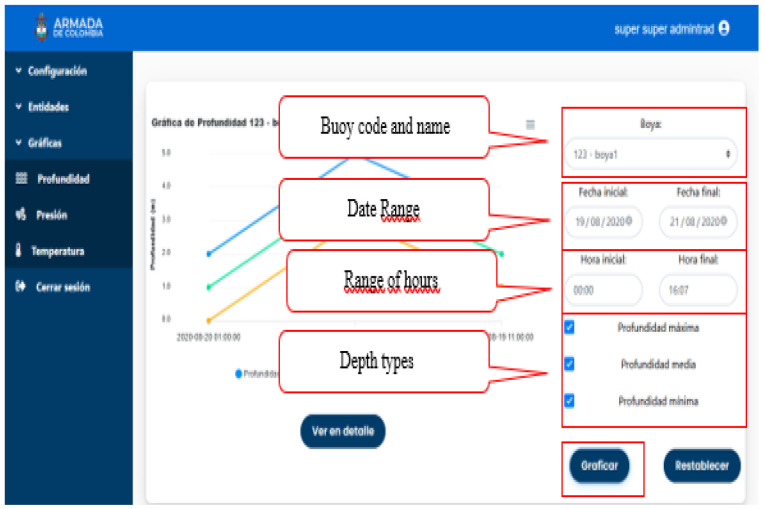
Buoys’ graphs.

In Figure 4 you can see the interaction of the software with the data from the buoys, making real-time reports of this information, which is consolidated by the following fields: name of the buoy, date range, range of hours, depth types.

### 4.3. Phase 3: Materials and Methods for Construction of the Predictive Model

In the first instance, it is necessary to indicate that the saline wedge or saline intrusion “arises when fresh water meets salty sea water and the first flows over the second due to the difference in densities. In other words, salt water, which is denser or heavier, penetrates below fresh water and displaces it due to the morphology of a riverbed, circulating flow, sea level, persistent winds, etc.” [29]. The intrusion of salt water into the estuary of the river generates sedimentation processes that hinder the navigability of ships depending on their draft, leading to an increase in the time that these ships must remain at sea, with subsequent economic implications. Therefore, with the intention of generating better navigability scheduling processes in the Magdalena River, it is necessary to predict in which periods of the year there is the presence of an exit wedge. Since salt or sodium chloride has ions that, when dissolved in water, form an electrically conductive solution, the parameter to monitor to detect the presence of saline wedge in the river estuary is the conductivity level in the water from the river.

To carry out such monitoring, within the framework of this project, two buoys have been installed in the cause of the Magdalena river, and in each one of them three sets of sensors were installed (fixed to the respective buoy at different underwater distances). Such sets of sensors measure the temperature, pressure, and conductivity of the water. From the above, a database was obtained for each of the three sets of sensors. This database contains information from 70 days of sample collection. Automatic data sensing has been carried out daily, in the time frame from 26 September 2019 to 4 December 2019, from 00:00 to 23:49. Predicting the presence and level of conductivity of the river water, in the time frame, would allow taking preventive actions in terms of scheduling the navigability processes of ships in the river. This influences the reduction of waiting times, in which ships remain anchored in the high seas, given that the presence of a saline wedge prevents the entry of such ships into the Magdalena River, due to the sedimentation effect caused by it.

The identification of a predictive model based on time series to detect the presence and the level of conductivity in river water involves two major stages: experimental and implementation. The main objective of the experimentation stage is the application of techniques based on data mining for the construction of the predictive model [30]; this requires: (1) the preparation and pre-processing of data; (2) identification, training and testing of the model; and (3) analysis of results, assessing performance quality metrics. Once the predictive technique has been identified and validated, based on time series, that allows the projection of conductivity levels in a future time frame, and we proceed to the implementation stage, in which it is necessary to identify the development framework. That is: (1) identification of the programming languages and environments that facilitate the integration of the predictive model with the technological solution; (2) connection of the model to the data source; and (3) development of the programming code that connects the model with the interface of user. For further illustration, this report details the two stages mentioned above.

The experimental process, as indicated above, requires the execution of the following activities: preparation and pre-processing of data, training, and testing of the model, and analysis of results (evaluating performance quality metrics).

#### 4.3.1. Data Preparation and Preprocessing

The data collected from the monitoring process were compiled into two files called Boya_3.csv and Boya_7.csv due to the name that was physically assigned to the buoys (which we will call raw data—raw). Said files have the following structure respectively, see Table 3:

Such files must be prepared and pre-processed to train and test the identified model. This implies: (1) pre-processing in terms of data quality and cleanliness and (2) organization of data collections (dataset).

#### 4.3.2. Cleanliness and Quality of Data

To homogenize the presentation of the data, the following actions were carried out:

- Leave only the data instances between the dates 26 September 2019 and 4 December 2019, in both CSV files.
- Delete from the Boya_3.csv file the column named TempInterna.
- Delete the columns in both CSV files: RF_IN, RF_OUT and Charging regulator.
- Make sure that both files contain the following columns of data: Date, time, Battery Voltage, Conductivity depth max, Conductivity depth medium, Conductivity depth min, Pressure depth max, Pressure depth medium, Pressure depth min, Temperature depth max, Temperature depth mean and min deep temperature (12 columns in total).
- Leave only the data instances sampled in minutes: 00, 15, 30, or 45.
- The other instances were removed because they contain null values.

#### 4.3.3. Organization of Data Collections

To organize the data collections (dataset), which will later be used in the training and testing process of the model, the following actions were carried out:

- Construction of TWO dataset from raw data, one for each buoy. Each will contain the average values of the instances per day. Those files were named: Boya3_full and Boya7_full.
- Construction of EIGHT dataset (Boya3_00_full, Boya3_15_full, Boya3_30_full and Boya3_45_full, in the same way for Boya7). These datasets will contain the respective averages per day of the instances, calculated from the time frames 00, 15, 30, and 45 min.
- Copy the EIGHT datasets above and only leave the columns: Date and Conductivity prof ma. Such files were named: Boya3_00_conduc, Boya3_15_conduc, Boya3_30_conduc, and Boya3_45_conduc, in the same way for Boya7.
- Create copies of the datasets built in the previous step and name them like this: Boya3_00_conduc will be called Boya3_00_conduc_train, Boya3_30_conduc will be called Boya3_30_conduc_train, Boya3_15_conduc will be called Boya3_15_conduc_test, and Boya3_15_conduc_test will be called Boya3_conduc45_duyactest_duyac45_test_duyac_test45_duyac_test_duyac45. These will be used for training and testing processes. Its distribution is 50% train and 50% test.

**Figure 5:**
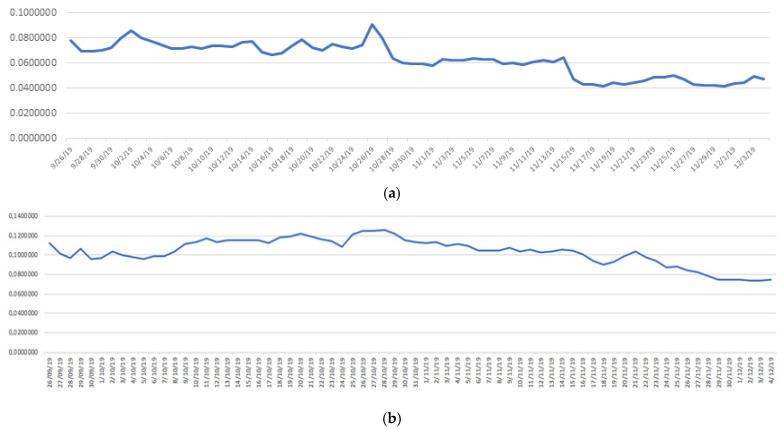
(**a**) Conductivity measurement on Buoy 3. (**b**) Measurement of conductivity in Buoy 7.

The EIGHT organized datasets have the following characteristics, see Table 4:

Figure 5 show the real behavior of conductivity, based on the data collected in buoys 3 and 7, respectively. Specifically, from the Boya_3_00_conduc_train.csv and Boya_7_00_conduc_train.csv files.

## 5. Contributions

In 1959, Arthur Samuel coined the term Machine Learning and defined it as “the field of study that gives computers the ability to learn without being explicitly programmed.” Machine learning is part of the field of Artificial Intelligence, and its objective is usually to recognize and fit statistics to models [31].

Along with Artificial Intelligence, Machine Learning has emerged as the method of choice for the development of practical software for image and speech recognition, natural language processing, robot control, and other applications. Many AI system developers recognize that, for many applications, it may be easier to train a system by feeding it examples of the desired input and output behavior, than to manually program in advance the desired response for all possible inputs [32].

Machine Learning has been playing, in recent decades, an important role in the construction of models based on experience from processed data [33], enabling computers to build models from data. For example, according to the problem to be solved and taking into account the input data [31,32,33], the construction of the appropriate algorithms is explored and studied, so that learning is achieved and making predictions from these dates.

As a result of the review of the scientific literature, regarding the implementation of techniques based on Machine Learning, for the approach of predictive solutions supported in time series, it was identified that the “Multi-Layered Perceptron” technique of the category Artificial Neural Networks has produced very good results in different fields of action, such as: investment models based on mutual funds [34,35], epidemiological models [35,36], estimation of the water recharge rate underground [37,38], analysis of the pedals interactions of race car drivers [39], efficient energy systems based on the prediction of natural gas consumption [40,41], and money flow prediction [42], among other studies.

The comparison of the scenarios is presented below, from the graphical analysis and from the evaluation of quality metrics (missing values, mean square error and prediction by scenario), all of them obtained after recreating the four experimentation scenarios, using the model based on “Multi-Layered Perceptron”. For the training and testing process, four experimentation scenarios have been recreated in which the datasets organized from the pre-processing of the previously explained data were used. The configuration of these deployment scenarios guarantees that we have two datasets with different data instances. Such scenarios are explained in Table 5:

### Graphical Analysis of the Behavior of the Model

The comparison of the scenarios is presented below, from the graphic analysis and from the evaluation of quality metrics (missing values, mean square error and prediction by scenario), all of them obtained after recreating the FOUR experimentation scenarios, using the model based on “Multi-Layered Perceptron”.

**Figure 6:**
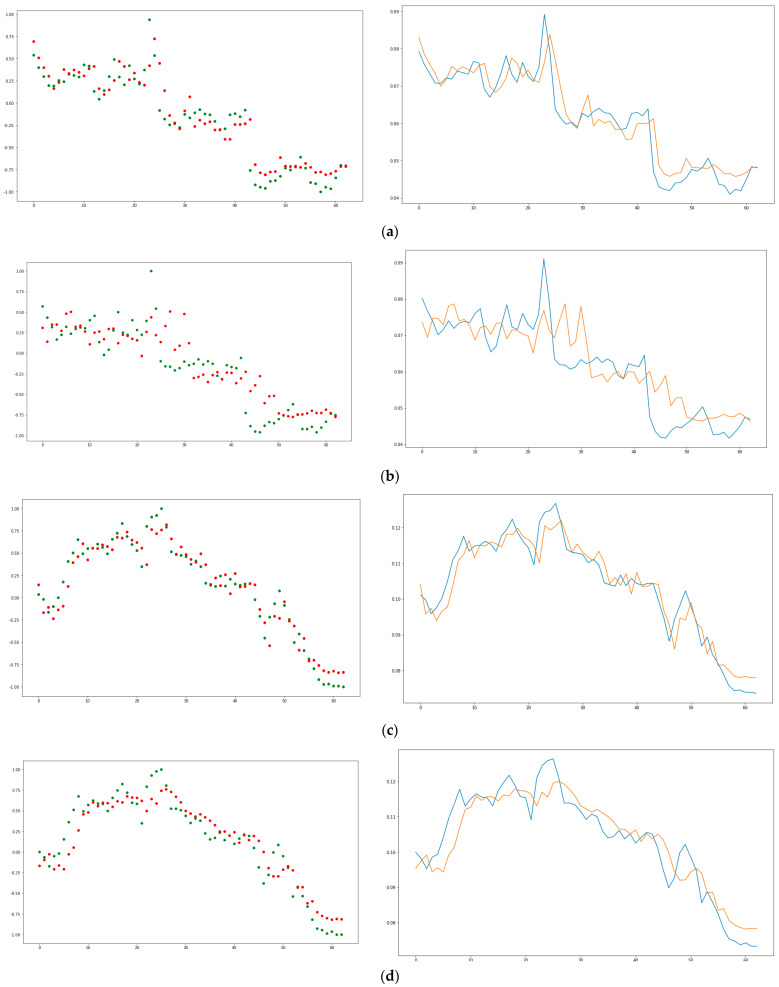
(**a**) Scenario 1 results. (**b**) Scenario 2 results. (**c**) Scenario 3 results. (**d**) Scenario 4 results.

The training and subsequent testing of the model is analyzed from the iteration of 40 EPOCHS. The point graphs (red points: prediction and green points: real) and lines (orange lines: prediction and blue lines: real) correspond to each experiment scenario recreated. The graphs show that the real conductivity level with the predicted conductivity level shows high similarity, see Figure 6. By graphically viewing the behavior of the data, the prediction model has made it possible to achieve good independent evaluation metrics of the data.

**Figure 7:**
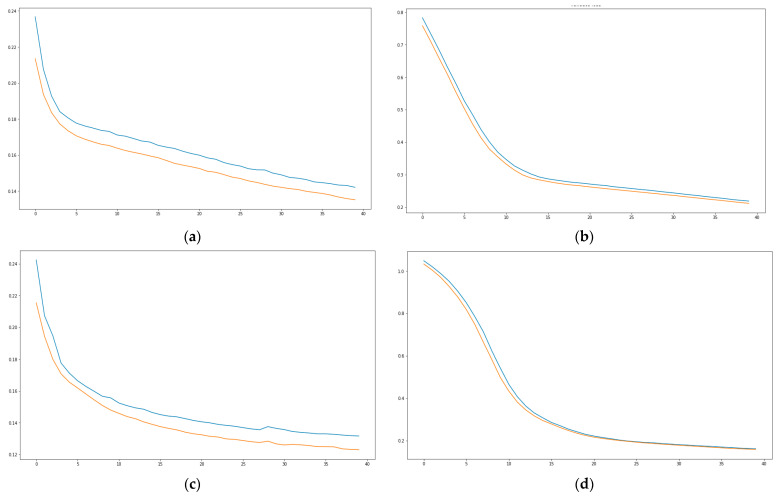
(**a**) Quality metrics Scenario 1, (**b**) Quality metrics Scenario 2, (**c**) Quality metrics Scenario 3, (**d**) Quality metrics Scenario 4.

Performing analyses based on quality metrics is carried out based on missing values, mean square error, and prediction by scenario. Regarding the analysis of the metric of missing values, the following graphs show that in all cases there has been a significant reduction of said values as the iterations of the 40 EPOCHS are executed, see Figure 7.

**Figure 8:**
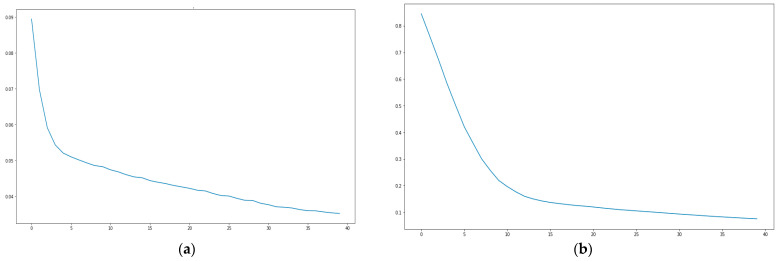

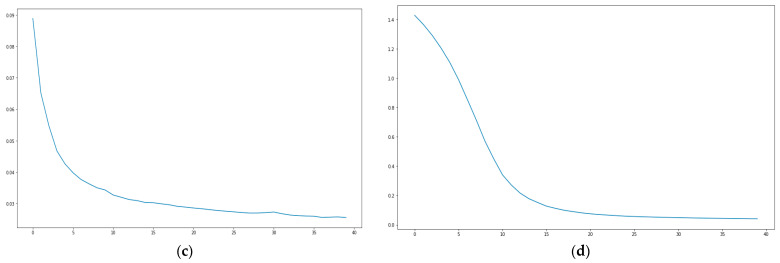
(**a**) Mean Square Error Scenario 1, (**b**) Mean Square Error Scenario 2, (**c**) Mean Square Error Scenario 3, (**d**) Mean Square Error Scenario 4.

Regarding the analysis of the mean square error, it is important to specify that this is inversely proportional to the accuracy of the model (accuracy). The following graphs show that during the iteration’s product of the 40 EPOCHS, there was a substantial reduction of the mean square error, which means that there is a significant increase in the predictive accuracy of the model, see Figure 8.

## 6. Conclusions and Future Works

**Figure 9:**
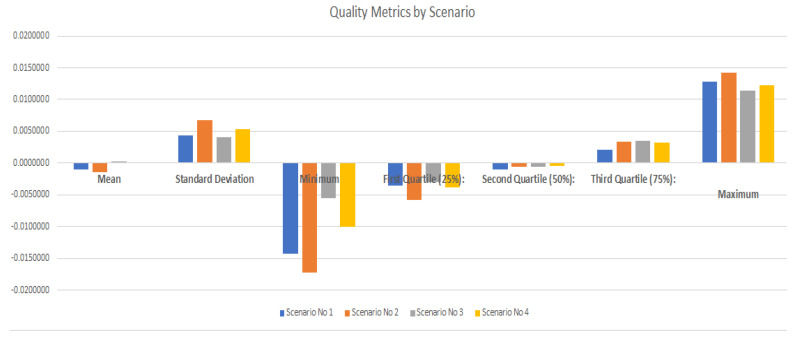
Quality metrics by scenario.

Various quality metrics were analyzed regarding the behavior of the predictive model by scenario. The results show consistency, see Table 6 and Figure 9:

The following table shows the predictions for 1 week (7 days), after the time frame of the data contained in the datasets. To analyze the reliability of the hypotheses, the analysis based on ANOVA was carried out, as shown in the Table 7. The results obtained are like the real data, see Table 8.

The results of the ANOVA table are described below:

1st species risk = Alpha = 0.05
Fisher table: F3.24 (5%) = 3.01–2419.48
Fisher table: F2.27 (5%) = 3.35–0.532

Therefore, there are no significant differences between the means, therefore the null hypothesis is accepted, which means that the results obtained in the predictions using the same model, in different sub-sets of data, are similar.

From the most relevant aspects that the development of this work brought about and generated innovation in the Colombian Caribbean region, the following can be highlighted:

- It is the first time that 24/7 monitoring with transmission in near real time of the saline intrusion in Bocas de Ceniza has been carried out, providing knowledge to port management from the point of view of maritime security.
- The analysis of saline wedge data versus other parameters will allow us to get closer to understanding the behavior of the river, perhaps predicting the behavior of sediment, thus giving an early warning of low draft in the port of Barranquilla.
- The navigable channel signaling system is used as an underwater monitoring station, optimizing the installed infrastructure.
- By the methodology used, we are knowing the speed of the saline intrusion in the Magdalena river, data that was not known.
- The information collected by the system will allow to significantly adjust any modeling to be carried out in the Magdalena River, improving the quality and precision of the predictions, by having a permanent validation source.
- It can be identified that when the iterations are increased, the quadratic error decreases and the accuracy increases.

The future work of this research is to extend the number of buoys that can provide information about the salt wedge phenomenon in the Magdalena River to validate this model with more data instances from the sensors. The increase in the number of sensors expands the coverage of the study area, which brings with it the validation of the model and the generation of new experimentation scenarios. These new experimentation scenarios in turn allow the use of new techniques, both in preprocessing and in the discovery of relationships between the study variables and comparison with the preliminary results.

## Figures and Tables

**Table 1:** Study area coordinates.

Latitude	Length
−74.819661 W	11.128976 N
−74.921967 W	11.128557 N
−74.921548 W	11.056439 N
−74.813092 W	11.056020 N

**Table 2:** Buoy location coordinates.

Geographical Position		
**Buoy 1**	LAT. −74.845796	LONG. 11.084671
**Buoy 6**	LAT. −74.838389	LONG. 11.058955

**Table 3:** File structures from buoys.

File	Boya_3.csv	Boya_7.csv
Fields (data columns)	Date, time, Battery Voltage, Conductivity depth max, Conductivity depth average, Conductivity depth min, Temperature Internal, Pressure depth max, Pressure depth average, Pressure depth min, RF IN, RF OUT, Charging Regulator, Temperature depth max, Temperature depth average and Temperature prof min.	Date, time, Battery Voltage, Conductivity depth max, Conductivity depth average, Conductivity depth min, Pressure depth max, Pressure depth average, Pressure depth min, RF IN, RF OUT, Charging Regulator, Temperature depth max, Temperature depth average and Temperature depth min.
Number of fields	16	15
Time frame	From 18 September 2019 at 12:06:17 pm to 5 December 2019 at 1:31:45 pm	From 25 September 2019 at 10:58:38 am to 5 December 2019 at 1:39:39 pm.
Number of instances (rows of data)	15.683	13.988

**Table 4:** Dataset Structure.

Files	Boya_3_00_conduc_train.csv Boya_3_30_conduc_train.csv Boya_7_00_conduc_train.csv Boya_7_30_conduc_train.csv	Boya_3_15_conduc_test.cvs Boya_3_45_conduc_test.cvs Boya_7_15_conduc_test.cvs Boya_7_45_conduc_test.cvs
Fields (data columns)	Date Maximum depth conductivity	Date Maximum depth conductivity
Number of fields	2	2
Time frame	From 09/26/2019 to 12/4/2019	From 09/26/2019 to 12/4/2019
Number of instances (rows of data)	70	70

**Table 5:** Details of the experimentation scenarios.

No. Scenario	No. Buoy	Training Dataset	Test Dataset
1	3	Boya_3_00_conduc_train.csv	Boya_3_15_conduc_test.cvs
2		Boya_3_30_conduc_train.csv	Boya_3_45_conduc_test.cvs
3	4	Boya_7_00_conduc_train.csv	Boya_7_15_conduc_test.cvs
4		Boya_7_30_conduc_train.csv	Boya_7_45_conduc_test.cvs

**Table 6:** Model quality metrics.

No. Scenario	Metrics	Real	Prediction	Scenario
1	Mean	0.0613490	0.0623860	−0.0010370
	Standard deviation	0.0126820	0.0118150	0.0043640
2	Mean	0.061465	0.062881	−0.001416
	Standard deviation	0.012894	0.010674	0.006691
3	Mean	0.103661	0.103459	0.000201
	Standard deviation	0.014309	0.013157	0.003998
4	Mean	0.103643	0.103588	0.000054
	Standard deviation	0.014406	0.012566	0.005294

**Table 7:** ANOVA analysis.

Origin of the Variation	Sum of Squares	Degrees of Freedom	Middle Square	F Ratio
Total	0.007651	27		
Treatments	0.007625612	3	0.002541871	2419.48221
Residual	0.000025	24	1.05058 × 10^−6^	

**Table 8:** One week predictions.

Maximum Depth Conductivity (mS/cm)				
Date	Scenario 1	Scenario 2	Scenario 3	Scenario 4
21/11/19	0.045417	0.044500	0.102292	0.102167
22/11/19	0.047667	0.045750	0.097958	0.098542
23/11/19	0.047167	0.046833	0.093708	0.094875
24/11/19	0.048208	0.048542	0.086833	0.085583
25/11/19	0.050667	0.050333	0.089375	0.088708
26/11/19	0.047667	0.047000	0.084458	0.085708
27/11/19	0.043667	0.042667	0.082000	0.082375
28/11/19	0.043333	0.042667	0.079083	0.078083
29/11/19	0.041000	0.043333	0.075792	0.075250
30/11/19	0.042333	0.041667	0.074417	0.074750
1/12/19	0.041917	0.043125	0.074583	0.073667
2/12/19	0.044917	0.044875	0.073958	0.074250
3/12/19	0.048375	0.047417	0.073917	0.073292
4/12/19	0.048083	0.046917	0.073667	0.073292
5/12/19	0.046493	0.042732	0.076291	0.075819
6/12/19	0.046408	0.043084	0.077089	0.076664
7/12/19	0.044692	0.043668	0.076857	0.076707
8/12/19	0.043904	0.043828	0.076837	0.077289
9/12/19	0.042995	0.042498	0.077129	0.077586
10/12/19	0.042284	0.044327	0.077053	0.077652
11/12/19	0.043292	0.045224	0.076775	0.077708

Fisher table: F2.27 (5%) = 3.35–0.532; Fisher table: F2.27 (5%) = 3.35–0.532.
